# The Journal of Therapeutic Ultrasound

**DOI:** 10.1186/2050-5736-3-S1-O65

**Published:** 2015-06-30

**Authors:** Wladyslaw Gedroyc, Robert Muratore

**Affiliations:** 1 Imperial College London, London, United Kingdom; 2 Quantum Now LLC, Huntington, New York, United States

## Background/introduction

Publications in therapeutic ultrasound have been growing exponentially for the past four decades, n = 2 exp((t-1970)/6), R² = 0.965, and reached approximately 1440 papers in 2011. Adoption of new International Electrotechnical Commission standards by regulatory agencies such as the United States Food and Drug Administration will enable more rapid approval of devices and therefore more clinical applications. Thus the exponential growth is likely to continue with a predicted output of 3600 papers by 2015. In order to serve this growing demand, the Journal of Therapeutic Ultrasound (JTU) has been launched. JTU is an online, open access journal designed to accelerate the adoption of focused ultrasound.

## Methods

The journal website [http://www.JTUltrasound.com] is currently accepting manuscripts of research articles, case reports, reviews, meeting reports, and study protocols. Appropriate topics include translational and clinical research in all areas of therapeutic ultrasound, including stimulation, inhibition, destruction, or modification of tissue function or structure via focused ultrasound. As an online journal, color images and supplemental materials such as audio and video files are readily accepted and publication rapidly follows review. As an open access journal, authors retain full rights to their papers through the Creative Commons license, and papers will be freely available and thus easily accessible to developing countries and the popular press.

## Results and conclusions

JTU serves as the official journal of the Focused Ultrasound Foundation and the International Society for Therapeutic Ultrasound. Our distinguished editorial board of twenty-six members represents ten countries. Author fees are waived through 2014.

